# Analisi della funzione tiroidea in 50 pazienti con COVID-19: uno studio retrospettivo

**DOI:** 10.1007/s40619-021-00948-8

**Published:** 2021-08-23

**Authors:** Nicola Viola, Francesco Latrofa

**Affiliations:** Dipartimento di Medicina Clinica e Sperimentale, Università di Pisa, Pisa, Italia

Commento a:

**Thyroid function analysis in 50 patients with COVID-19: a retrospective study.**

**M. Chen, W. Zhou, W. Xu.**

**Thyroid (2021) 31(1):8–11**

L’infezione da SARS-CoV-2, responsabile della COVID-19, ha interessato oltre 200 paesi nei 6 diversi continenti. Sebbene sia ormai noto che si tratti di una malattia con coinvolgimento multi-organo, al momento non è stato chiaramente definito l’impatto della COVID-19 sulla funzione tiroidea.

Lo scopo di questo studio è stato quello di valutare retrospettivamente la funzione tiroidea in pazienti con COVID-19. Tale studio ha riguardato 50 individui cinesi affetti da COVID-19 e privi di storia di malattie della tiroide nei quali sono stati valutati i livelli sierici di tiroxina totale (TT4), triiodotironina totale (TT3) e ormone stimolante la tiroide (TSH) nel corso di COVID-19 e dopo la guarigione. I gruppi di controllo erano costituiti da soggetti sani e pazienti con polmonite non sostenuta da SARS-CoV-2. Nei pazienti affetti da COVID-19 sono stati riscontrati livelli inferiori sia di TT3 che di TSH rispetto ai controlli, mentre non è stata rilevata alcuna differenza riguardo i valori di TT4. Inoltre, gli autori hanno osservato una correlazione inversa tra la gravità del COVID-19 e i livelli di TT3 e TSH. Dopo la guarigione non è stata osservata alcuna differenza nei test di funzionalità tiroidea tra i pazienti con COVID-19 e quelli dei gruppi di controllo. Questi risultati sono indicativi della presenza di una *non-thyroidal illness syndrome* (NTIS) tipica di molti stati patologici con coinvolgimento sistemico e che si risolve con la guarigione.

Tuttavia, il fatto che i livelli sierici di TSH dei pazienti con COVID-19 fossero significativamente più bassi rispetto ai pazienti con polmonite non COVID-19 con un simile grado di gravità potrebbe indicare un’inibizione nei confronti delle cellule ipofisarie TSH-secernenti mediante un meccanismo virale diretto o mediato dall’attivazione di varie citochine proinfiammatorie o legato alle alterazioni dei feedback ormonali indotti dai trattamenti farmacologici, come i glucorticoidi assunti da 31/50 dei pazienti con COVID-19 valutati in questo studio.

